# P11 Fluoroquinolone MHRA alert—how we reduced our fluoroquinolone use by half

**DOI:** 10.1093/jacamr/dlaf118.018

**Published:** 2025-07-14

**Authors:** K Atack, S Hackney, L Nguyen, Z Tariq, S Nazir, E O’Cofaigh

**Affiliations:** Leeds Teaching Hospitals Trust (LTHT), Leeds, UK; Leeds Teaching Hospitals Trust (LTHT), Leeds, UK; Leeds Teaching Hospitals Trust (LTHT), Leeds, UK; Leeds Teaching Hospitals Trust (LTHT), Leeds, UK; Leeds Teaching Hospitals Trust (LTHT), Leeds, UK; Leeds Teaching Hospitals Trust (LTHT), Leeds, UK

## Abstract

**Background:**

The Medicines and Healthcare Regulatory Agency (MHRA) released a Drug Safety Update in January 2024 advising the limitation of the use of fluoroquinolones due to irreversible and lifelong debilitating side effects. As part of the action plan at Leeds Teaching Hospitals NHS Trust (LTHT), all guidelines containing fluoroquinolones were identified and sent to the Infection Specialist teams to review whether they could be changed to an alternative, or whether a fluoroquinolone would need to remain.

**Objectives:**

To review all guidelines containing fluoroquinolones and replace them where possible and to reduce the amount of fluoroquinolones prescribed across the organization.

**Methods:**

All guidelines were sent to Infection Specialists and reviewed. Once decisions had been made whether to keep fluoroquinolones in the guidelines, or use an alternative, the guidelines were sent out for peer review for approval from the Improving Antimicrobial Prescribing Group (IAPG). Once approved, the guidelines were updated with any changes and the review was briefed across the organization. Additionally, when the alert was first published, communications were sent across the organization immediately to highlight the content of the alert.

**Results:**

There were 122 mentions of fluoroquinolones, advised across 49 paediatric and adult antimicrobial guidelines. Fluoroquinolones were changed to alternatives in 55 circumstances. Most common switches were to gentamicin, aztreonam and co-trimoxazole. Defined daily doses of fluoroquinolones were >600 in January 2024 at the time the alert was published. This has dropped to just above 300 as of February 2025.

**Conclusions:**

Interventions including communications throughout the organization via digital messages such as e-mails and bulletins, updates to the electronic medicines chart and information on our antimicrobial guidelines website to raise awareness of the MHRA alert and highlight to clinicians the conditions to avoid using fluoroquinolones in were deployed immediately after the alert was published. The review of the fluoroquinolone guidelines was a longer piece of work that required collaboration between Infection Specialists to identify where fluoroquinolones could be replaced with effective and safer alternatives. These decisions were made based on expert knowledge, national guidance and resistant patterns across the local area. The communications and review of guidelines has led to a significant and sustained reduction in fluoroquinolone prescribing over the last year. For every new guideline or guideline review, the use of fluoroquinolones will be carefully considered, taking into account the MHRA alert information.

